# Development and validation of the Treatment Related Impact Measure of Weight (TRIM-Weight)

**DOI:** 10.1186/1477-7525-8-19

**Published:** 2010-02-05

**Authors:** Meryl Brod, Mette Hammer, Nana Kragh, Suzanne Lessard, Donald M Bushnell

**Affiliations:** 1 The Brod Group, 219 Julia Avenue, Mill Valley, California 94941, USA; 2 Novo Nordisk A/S, Global Development, Krogshøjvej 29, 2880 Bagsværd, Denmark; 3 Health Research Associates, 6505 216th Street SW, Suite 105, Mountlake Terrace, Washington 98043, USA

## Abstract

**Background:**

The use of prescription anti-obesity medication (AOM) is becoming increasingly common as treatment options grow and become more accessible. However, AOM may not be without a wide range of potentially negative impacts on patient functioning and well being. The Treatment Related Impact Measure (TRIM-Weight) is an obesity treatment-specific patient reported outcomes (PRO) measure designed to assess the key impacts of prescription anti-obesity medication. This paper will present the validation findings for the TRIM-Weight.

**Methods:**

The online validation battery survey was administered in four countries (the U.S., U.K., Australia, and Canada). Eligible subjects were over age eighteen, currently taking a prescription AOM and were currently or had been obese during their life. Validation analyses were conducted according to an *a priori *statistical analysis plan. Item level psychometric and conceptual criteria were used to refine and reduce the preliminary item pool and factor analysis to identify structural domains was performed. Reliability and validity testing was then performed and the minimally importance difference (MID) explored.

**Results:**

Two hundred and eight subjects completed the survey. Twenty-one of the 43 items were dropped and a five-factor structure was achieved: Daily Life, Weight Management, Treatment Burden, Experience of Side Effects, and Psychological Health. *A-priori *criteria for internal consistency and test-retest coefficients for the total score and all five subscales were met. All pre-specified hypotheses for convergent and known group validity were also met with the exception of the domain of Daily Life (proven in an ad hoc analysis) as well as the 1/2 standard deviation threshold for the MID.

**Conclusion:**

The development and validation of the TRIM-Weight has been conducted according to well-defined principles for the creation of a PRO measure. Based on the evidence to date, the TRIM-Weight can be considered a brief, conceptually sound, valid and reliable PRO measure.

## Introduction

The use of prescription anti-obesity medication (AOM) to treat obesity is becoming increasingly common as treatment options grow and become more accessible. However, AOM has been associated with a wide range of potentially negative impacts on patient functioning and well being. Unfortunately, the impact of AOM is far less well understood than the impact of obesity on Health Related Quality of Life (HRQoL). The main challenge in understanding these impacts is the absence of a conceptual and psychometrically sound treatment-specific measure to assess the full range of key impacts of anti-obesity medication treatment on all aspects of patients' lives.

Patient-reported outcomes on weight management are thus especially important since patients may use different criteria than practitioners to assess treatment efficacy with respect to weight loss, improvement in co-morbidities and changes in quality of life. For example, patients often have unrealistic expectations regarding weight loss treatments, and may have a clinically significant amount of weight loss, but remain dissatisfied [1,2]. Treatment satisfaction may be correlated with patient compliance [3-5], impaired self-management [6], health care decisions [7], and use of health care services [8]. It is also associated with improvements in treatment efficacy outcomes [9], and patients who are satisfied with their treatments are more likely to maintain positive physical and psychological health [10]. Therefore, assessing treatment satisfaction can help the physician distinguish among treatment regimes with equal efficacy or impact on HRQoL [11], as well as identify treatments that patients find more acceptable [10], potentially resulting in greater compliance and thereby efficacy. Finally, both side effects and treatment burden seem to drive many of the negative impacts in the other domains, resulting in poor treatment compliance, leading to further decreasing drug efficacy and treatment satisfaction [5,12-14].

**Figure 1:**
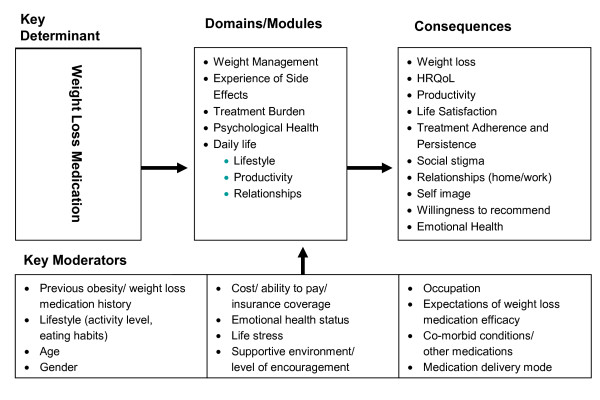
**Theoretical Model**.

The Treatment Related Impact Measure (TRIM-Weight) is an obesity treatment-specific patient reported outcomes (PRO) measure designed to assess the key impacts of prescription anti-obesity medication and be applicable to the wide range of prescription medications currently available [12,15]. The TRIM-Weight was developed following the draft Food and Drug Administration (FDA) guidelines for the development of patient reported outcome (PRO) measures, including patient focus groups and item generation based on a conceptual model [16]. Treatment-specific measures, based on input from clinical experts and patients with the condition of interest, are more targeted to a specific patient population and incorporate only issues of relevance to that population. In order to fully understand the impact of AOM in obesity, data were collected from three sources: literature, clinical experts, and respondents in three countries (U.S., U.K. and France). Focus groups were held in five cities in the three countries (Dallas, Chicago, Los Angeles, London and Paris). Nine focus groups were required to reach saturation of information, both within and between countries, whereby no new information was generated. A total of 70 eligible respondents participated in the focus groups (29 men [11 U.S., 10 U.K. and 8 France] and 41 women [25 U.S., 8 U.K. and 8 France]). Analysis of the interview transcripts identified five hypothesized domains that were most impacted by AOM: Psychological Health, Daily Life, Treatment Burden, Weight Management, and Experience of Side Effects and a theoretical model of the impact of AOM on patient functioning and well-being was developed (Figure 1).

Based on this theortetical model, and relying primarily on the wording of impacts used by patients, items were generated for each of the conceptual model domains. These items then underwent cognitive debriefing in an independent sample of obese adults who were recruited and met the same eligibility criteria as the interview sample to assess readability, comprehension of intended meaning, and relevance. A validation ready version of the TRIM-Weight was then developed. This paper will present the validation findings for this obesity prescription AOM-specific PRO measure, the TRIM-Weight.

## Methods

### Procedures

The debriefed version of the TRIM-Weight was incorporated into an online validation study to assess the measurement and psychometric properties of the measure. As with the development phase, the validation study methodology closely followed the guidelines laid out by the FDA for the development of a PRO measure [16]. Institutional Review Board approval was obtained for the study and all participants provided informed consent.

The validation battery survey was administered in three countries (the U.S., Australia, and Canada) to a sample independent of the development sample. Subjects eligible for the study were over age eighteen, currently taking a prescription AOM and were either currently or had been obese during their life (BMI between 30 and 45). Two recruitment strategies were employed to recruit the validation sample. The primary strategy was to identify eligible subjects in the U.S., U.K., Canada and Australia via a database of subjects who had previously agreed to be contacted for research purposes, managed by an academic unit of The University of Syracuse. Eligibility was assessed online for the sample based on self-reported responses to screening questions. Those passing the screening questions were then allowed into the survey. Additional participants were recruited by an advertisement on Craig's List, a U.S. national network of online communities. For the Craig's List sample, those responding to the advertisement were screened by telephone. Respondents who were eligible and willing to participate were emailed the URL link to access the validation survey and provided a unique ID number. Regardless of recruitment strategy, all data management and maintenance of the survey was conducted by the first author.

### Measures

In conjunction with the validation version of TRIM-Weight, several additional measures were included in the study and chosen for their comparative value for this validation study, their high level of established validity, and brevity in their administration. These measures include the following:

#### Center for Epidemiologic Studies Depression Scale (CES-D)

This measure includes twenty items comprising six scales reflecting major dimensions of depression: depressed mood, feelings of guilt and worthlessness, feelings of helplessness and hopelessness, psychomotor retardation, loss of appetite, and sleep disturbance experienced in the past week. Higher scores (both item and total scores) indicate more depressive symptoms. An average score of 16 or higher on this scale suggests that the population under study incurs a high risk for depression [17]. Introduced and validated in 1977, this measure has been used extensively as a research measure ever since. The original 1977 validation research for this measure demonstrated an internal consistency ranging from .85 to .90 (coefficient alpha and Spearman-Brown, split halves method) [17]. The test-retest reliability was in the moderate range for all time intervals, ranging from .45 to .70, with the author's assessment of the "fairest" estimate of test retest reliability as r = .54 [17].

#### Patient Health Questionnaire 15-Item Somatic Symptom Severity Scale (PHQ-15)

This 15-item somatic symptom subscale of the Primary Care Evaluation and Mental Disorders (PRIME-MD) is a diagnostic instrument for common mental disorders. Internal reliability is high, with a Cronbach's alpha of .80 [18]. Convergent and discriminant validity was established in a two-sample study comprising 6000 participants [18]. In a more recent study, the sensitivity (78%), specificity (71%), and test-retest reliability (.60) established the PHQ-15 as valid and "moderately reliable" in detecting somatoform disorders [19]. The PHQ-15 measures somatic symptom severity [18].

#### The SF-12v2™ Health Survey

The SF-12v2 is a 12-item instrument for measuring health status and outcomes from the patient's point of view in each of eight health concepts: physical functioning, role limitations due to physical health problems, bodily pain, general health, vitality (energy/fatigue), social functioning, role limitations due to emotional problems and mental health (psychological distress and psychological well being). A high score indicates a more favorable health state [20]. Derived from the longer SF-36 Health Survey, the short form uses two of the longer survey's components, the Physical Component Summary (PCS) and the Mental Component Summary (MCS). The SF-12 demonstrates multiple R squares of 0.911 in prediction of the SF-36 PCS and 0.918 in prediction of the SF-36 MCS. In the general population, it achieved R squares of 0.905 and 0.938 for the PCS and MCS, respectively. Two-week test retest correlations of 0.89 were observed for the PCS and 0.76 for the MCS. Furthermore, it has been validated for populations beyond the United States [21]. Last, version 2 (the version utilized in this study) is valid and demonstrates high internal consistency reliability with alpha > 0.80 and a high test-retest reliability for the PCS of intraclass correlation coefficient of 0.78. The MCS demonstrates a moderate test-retest reliability of intraclass correlation coefficient of 0.60 [22].

#### Activity Impairment Assessment (AIA)

This five-item questionnaire assesses the amount of time that an individual's work or regular activities have been impaired as a result of their condition. Responses are provided in a 5-point Likert-type scale format, ranging from "none of the time" to "all of the time," and given a score ranging from 0-4. The questionnaire is scored for the total score [23]. The AIA has a high level of internal consistency with Cronbach's alpha = 0.93. It also has high levels of convergent validity (all r_s _> 70), and divergent validity (r_s _= .078). Excellent discriminant validity has been demonstrated in relation to clinical evaluations [23].

#### Insulin Treatment Satisfaction Questionnaire (ITSQ)

The ITSQ is a 5 factor, 22-item questionnaire that discerns treatment satisfaction for diabetic patients who are using insulin. In addition to an overall score, the items comprise five domains: inconvenience of regimen, lifestyle flexibility, glycemic control, hypoglycemic control, and insulin delivery device satisfaction. A higher score indicates greater satisfaction with treatment. Only the inconvenience of regimen domain, which is not specific to diabetes, was used in this study [10]. In total, the ITSQ demonstrates an internal consistency (using Cronbach's alpha coefficient) of the subscales ranging from 0.79 to 0.91. Additionally, test-retest reliability (using Spearman rank correlation coefficients) ranged from 0.63 to 0.94. These scores show moderate to high correlation with related measures of treatment burden [10].

#### Treatment Satisfaction Questionnaire for Medication (TSQM)

This is a fourteen-item questionnaire that measures a patient's experience with medication in terms of four scales: side effects, effectiveness, convenience, and global satisfaction. A higher score indicates greater satisfaction with treatment [24]. In a validation study centered on a variety of chronic diseases, factor analysis demonstrated three factors (eigenvalues > 1.7) explaining 75.6% of total variance. These factors, using Cronbach's alpha coefficient, ranged from 0.85 to 0.87. An additional factor analysis yielded a Global Satisfaction Scale which, using Cronbach's alpha coefficient, demonstrated a consistency of 0.85 [24]. The TSQM-9 also demonstrates good test-retest reliability with intraclass correlation coefficients > 0.70 [25].

#### Frequency, Intensity, and Burden of Side Effects Rating (FIBSER)

This three-item questionnaire measures medication side effect impact over the past week using three domains: frequency, intensity, and burden (the degree that medication interfered with day-to-day functions). The FIBSER was shown to have high levels of internal consistency with Cronbach's alpha values ranging from 0.91 to 0.93 over multiple assessments of participants' side effects experiences [11]. The FIBSER was also shown to be reliable (with high correlations between observations made a short time apart), sustaining correlations at Week 4 (with Week 2) of 0.46 (frequency), 0.48 (intensity), and 0.45 (burden) [26]. The FIBSER has shown significant construct validity (p < 0.0001) [26].

#### Quality of Life Enjoyment and Satisfaction Questionnaire (Q-LES-Q) (Short Form)

Used widely to measure patient satisfaction pre- and post- treatment, this 16-item questionnaire assesses the degree of enjoyment and satisfaction experienced in eight areas: physical health, subjective feelings of well being, work, household duties, school, leisure, social relationships, and general life quality. Scores are aggregated, with higher scores indicative of greater enjoyment or satisfaction in each domain [27]. In a 2007 study of control volunteer subjects, the Q-LES-Q demonstrated high internal consistency, with coefficients for each domain ranging from 0.82 to 0.90. Intraclass coefficients for these domains ranged from 0.58 to 0.89 [28].

#### Medication Compliance Scale (MCS)

A six item measure assessing how often a patient thinks about postponing or skipping doses, or has actually postponed or missed doses over the past two weeks. Items are scored on a six-point Likert scale, from 0 (never) to 5 (always). The total score is calculated by summing item values, with a higher score indicating poorer compliance. This measure has not yet been validated [6]. Although this measure is currently not validated, it was chosen due to its high face validity and proven ability to differentiate known groups in validation studies of other PRO measures [29].

### Statistical Strategy

Validation analyses were conducted according to an *a priori *developed statistical analysis plan (SAP). First, item level psychometric and conceptual criteria were used to refine and reduce the preliminary item pool and reduce redundancy between items. Next, factor analysis to identify structural domains was performed. Reliability and validity testing were then performed. To assess reliability, internal consistency and test-retest reliability were examined. To assess validity, content and construct validity (convergent and known-group) were examined. It is the intention of the developers that the TRIM-Weight can be used either as a total score or that each domain could stand alone as a separate measure. Therefore, all reliability and validity tests were performed on both the total score and for each domain. All data analyses were conducting using SPSS [30].

### Analysis Plan

To assess item characteristics and the measurement model (scaling) for the measure, the following tests were performed:

#### Item reduction

For item reduction, both item psychometric properties and conceptual importance were taken into consideration in making retention/deletion decisions for the initial item pool. Items were considered for deletion, based on psychometric criteria, if the item had missing data (i.e., no response) >5% of the time, if ceiling effects were present (>50% optimal response) or if item-to-item correlations within the total item pool were high, thus indicating redundancy between items (Pearson's correlation coefficient >0.70) [31]. Items that did not perform well psychometrically could be considered for retention if conceptually important and/or unique, but were otherwise dropped.

#### Factor structure

Factor structure was determined by an exploratory factor analysis using a Varimax orthogonal rotation with Kaiser normalization. The number of factors was not specified. Item-to-total scale correlations were assessed using the Pearson's correlation between individual item scores and the total subscale score for the associated subscale. Correlation coefficients < 0.40 were considered evidence of poor association [32]. The most appropriate number of factors to be extracted was determined by both the residual analysis, i.e., evaluation of the ability of the factor solution to represent the correlation structure, using 0.40 as the minimum factor loading to be eligible as an item for a given factor, as well as taking into consideration the clinical and theoretical interpretability of the solution. A scree plot of the principle component solution was used as guidance to the number of components with eigenvalues of greater than one.

To confirm the factor structures and to test the fit of the domains, a confirmatory factor analysis was performed using Mplus (Version 5.21). The Comparative Fit Index (CFI) was examined for model fit with a threshold of ≥ 0.90 indicating acceptable fit [33].

#### Reliability

Internal consistency reliability was examined using Cronbach's alpha statistics for the TRIM-Weight total and subscale scores. An alpha of > 0.70 was considered evidence of acceptable internal consistency [31,34].

Test-retest reliability was assessed at approximately two weeks post initial completion of the battery. To be eligible for the retest, participants had to respond "No" to the questions: "Have you experienced any major life events since you filled out the previous questionnaire approximately 2 weeks ago (e.g., moving, divorce, losing job)?" and "Has the past 2 weeks been an unusually stressful period for you?" and respond "Yes" to the question: "Have you been taking the same prescription weight loss medication over the past 2 weeks?" Reproducibility was assessed using the intraclass correlation coefficient (ICC). An ICC of >0.70 was considered evidence of acceptable test-retest reliability [31].

#### Convergent Validity

Convergent validity was evaluated by testing the following *a priori *defined hypotheses using a two-tailed test at a p < 0.05 level. When more than one hypothesis per domain is proposed, the minimum threshold of one hypothesis had to be met to claim convergent validity. The hypotheses were:

H_01_: For the total score there will be a correlation with Life Satisfaction (QLES) and/or the self-report overall item.

H_02_: For the Psychological domain there will be a correlation with Mental Health (SF-12) and/or the self report overall Psychological Health item.

H_03_: For the Daily Life domain there will be a correlation with Impairments in Activities (AIA) and/or self report overall life impact item.

H_04_: For the Burden domain there will be a correlation with Treatment Burden (TSQM domain) and Inconvenience (ITSQ domain) and/or self report overall item.

H_05_: For the Side Effects domain there will be a correlation with Side Effect Frequency/Severity (FIBSER) and/or self report overall side effects item.

H_06_: For Efficacy (Weight Management) there will be correlations with Treatment Efficacy (TSQM domain) and/or self report overall efficacy item.

#### Criterion Validity

Criterion validity is a measure of how well one variable or set of variables predicts an outcome. Criterion validity was tested by *a priori *hypotheses evaluating known-group for each domain and the total score. The scores of the groups on the TRIM-Weight domains were compared using one-way ANOVA with groups as a fixed factor. When more than one hypothesis per domain is proposed, the minimum threshold of one hypothesis had to be met to claim known-group validity. The hypotheses were:

H_07_: For the total score, those with higher total score will be more willing to stay on their AOM for a greater period of time and/or be more compliant with their AOM.

H_08_: For the Psychological domain, those with a higher score will have less depression and/or self report more supportive spouse/friends regarding weight loss.

H_09_: For the Daily Life domain, scores will be lower for those who work and/or those who have larger families.

H_10_: For the Burden domain, those who have to take multiple tablets per day will have greater domain scores.

H_11_: For the Side Effects domain, those with greater somatization scores will have a greater domain score.

H_12_: For the Efficacy (Weight Management) domain, those who report on average more weight loss per length of time on drug will have greater efficacy.

#### Interpretability

To assess interpretability, the minimal important difference (MID) was examined. To calculate the MID, the relationship and magnitude of change between these self-report "overall" items to the scores of each TRIM-Weight domain score were examined. The MIDs considered changes in scores of TRIM-Weight domains between responses of "A little" and "Somewhat" as the minimally important interval. For example, the difference in the mean response for the TRIM-Weight Burden domain score for those who respond "A little" and those who respond "Somewhat" on the independent item: "Overall, how inconvenient is your weight loss medication?" was calculated. For the total score, the difference between the "No impact at all" and "Slightly positive impact" response categories was examined. One-half standard deviation was considered the threshold difference for the MID.

## Results

### Item Generation and Cognitive Debriefing

The items were generated based on the conceptual model and worded to closely match patient statements. Examples of patient statements and corresponding items per domain are shown below. These items then underwent cognitive debriefing. Four iterations (three blocks of three participants and one block of two for a total of eleven adults, four men and seven women) were required to refine the items in terms of readability, relevance, and formatting and reach consensus in an entire block. As a result of the cognitive debriefing, a 43-item TRIM-Weight was generated.

### Validation Study

#### Sample

Via the primary strategy to find eligible subjects a total of 195 subjects entered the Study Response survey portal for the online validation survey; two subjects did not agree to take the survey after signing in and were exited from the survey. Thirty-two subjects agreed to complete the survey, but did not meet BMI eligibility requirements. Of the remaining 161 subjects, ten were not eligible, as they were not currently taking a prescription an anti-obesity medication. Finally, one subject stopped answering the items before getting to the TRIM-Weight items. From the second strategy, a total of fifty-nine subjects entered the Craig's List survey portal; only one did not answer any questions, leaving a total of fifty-eight completed surveys. The combined final sample for validating the TRIM-Weight was comprised of 208 subjects and is shown in Table 1.

**Table 1:** Validation Study Sample Description

Demographics Characteristics	Total N = 208
GENDER	

- Female	163 (78.4%)

- Male	44 (21.2%)

AGE (Years):	

- Mean (Std. Deviation)	38.2 (10.3) years

- Range	20-76 years

Weight* (current, in kg [lbs]):	

- Mean (Std. Deviation)	91.1 [200.8] (42.2)

- Range	54 [120]-147 [325]

BMI (at highest weight):	

- Mean (Std. Deviation)	36.4 (4.4)

- Range	30-45

TYPE OF OAM MEDICATION (% of sample)	

Phentermine	43.3%

Phendimetrazine	3.8%

Sibutramine	22.1%

Diethylpropion	3.4%

Orlistat	23.1%

Other/Missing	4.4%

EDUCATION:	

- Less than or Completed High School or GED	84 (41.61%)

- College Degree (Associate's Degree or B.A.)	96 (47.5%)

- Graduate Degree (or higher)	22 (10.9%)

ETHNICITY:	

- White/Caucasian	168 (83.2%)

- Black/African American	14 (6.9%)

- Latino/Hispanic/Mexican American	10 (5.0%)

- Native American/Alaskan Native	1 (0.5%)

- Asian American/Pacific Islander	5 (2.5%)

- Mixed Racial Background	2 (1.0%)

- Other Races	2 (1.0%)

CURRENT LIVING ARRANGEMENT:	

- Living with a spouse (% Yes)	169 (81.3%)

- Do you have children (% Yes)	50 (24.0%)

EMPLOYMENT:	

- Full-time paid position	119 (59.8%)

- Part-time paid position	23 (11.6%)

- Not currently working for pay	47 (23.6%)

- Student	10 (5.0%)

HOUSEHOLD INCOME	

- Less than $20,000	15 (7.4%)

- $20,000 to $39,999	32 (15.8%)

- $40,000 to $59,999	45 (22.3%)

- $60,000 to $79,999	50 (24.8%)

- $80,000 to $99,999	29 (14.4%)

- $100,000 and over	30 (14.9%)

- Declined to answer	1 (0.5%)

^1 ^One observation missing

^2 ^One observation was deleted as out of range.

#### Analysis

##### Item reduction

Twenty-one of the 43 items were dropped due to redundancy with other items, ceiling effects, poor factor loadings and/or did not fit conceptually with other items in the domain or did not tap highly relevant concepts based on patient reported information collected in the development phase. This resulted in a 22-item measure, which was used for the remaining analyses.

##### Factor structure

As hypothesized in the SAP, a five-factor structure, reflecting the hypothesized domains, was achieved with six items making up the Daily Life domain (component regression coefficients range .608 - .796): three items in Weight Management (component regression coefficients range .729 - .805), four items in Treatment Burden (component regression coefficients range .646 - .729), five items in Experience of Side Effects (component regression coefficients range .475 - .758), and four items making up the Psychological Health domain (component regression coefficients range .661 - .776). The scree plot confirmed five factors with eigenvalues of greater than one.

The domains were confirmed with CFI values all above 0.90: Daily Life, 0.977; Weight Management, 1.000; Treatment Burden, 0.996; Side Effects, 0.961; Psychological, 1.000; and Total, 0.930.

##### Reliability

As seen in Table 2, internal consistency, as measured by Cronbach's alpha of the TRIM-Weight Total score and all five subscales ranged between 0.71 and 0.94. The ICC values for test-retest reliability ranged from 0.75 to 0.86. This met the a priori hypotheses regarding internal consistency and reproducibility.

**Table 2:** Reliability Statistics on the TRIM-Weight

TRIM-Weight Domain	Internal Consistency Reliability (Cronbach's alpha)	Test-Retest Reliability N = 75 (ICC)
TRIM-Weight Total	0.9389	0.8554

Daily Life	0.9199	0.7588

Weight Management	0.7076	0.7527

Treatment Burden	0.7496	0.7699

Experience of Side Effects	0.8829	0.7554

Psychological Health	0.8799	0.7798

##### Convergent Validity

All pre-specified hypotheses were met at p < 0.001. The Total TRIM-Weight significantly correlated (r = 0.62) with the overall life satisfaction scale of the Q-LES-Q and the Psychological Health subscale (TRIM-Weight) had a significant association with the SF-12 mental component summary (r = 0.60). The Daily Life subscale correlated significantly with the AIA total score (r = 0.74), while the Treatment Burden subscale had a correlation of 0.70 with the TSQM-Burden. Finally, predictions were met regarding strong correlations between the Experience of Side Effects subscale and the FIBSER total score (0.74).

Significant correlations were found between all of the self-report overall items and their respective domains or total score. Specifically, the TRIM-Weight Total score was significantly correlated with the item "Overall, how much of an impact has your weight loss medication had on your life?" (r = 0.43). The Daily Life domain was significantly correlated with the item "Overall, how much does your weight loss medication impact your daily life?" (r = 0.47). For the Weight Management domain, there was a significant correlation with the item "Overall, how well does your weight loss medication work?"(r = 0.63). The Treatment Burden domain was significantly correlated with the item "Overall, how convenient is your weight loss medication?" (r = 0.64). There were also significant correlations for the Side Effects domain with the item "Overall, how much do side effects from your weight loss medication negatively impact you?" (r = 0.68) and for the Psychological Health domain with the item "Overall, how much does your weight loss medication negatively impact your psychological health?"(r = 0.55).

##### Criterion Validity

The specified *a priori *tests for known-group validity were met for the total score and all domains, with the exception of the domain of Daily Life, which was proven in an ad hoc analysis. The total TRIM-Weight was able to distinguish between groups likely or not likely to recommend their current treatment to a friend (F = 26.69, p < 0.001). There was also a significant difference between those compliant versus those not being compliant with their treatment (F = 52.60, p < 0.001). The total TRIM-Weight was not able to discriminate the length of time willing to stay on the current treatment, as this was likely confounded by how long the patients had already been on their treatment. The Psychological Health subscale was able to discriminate between depression severity (F = 77.41, p < 0.001) and level of social support from both family (F = 2.29, p < 0.05) and friends (F = 4.43, p < 0.05). The Treatment Burden subscale significantly differentiated treatment frequency coded as one time a day, twice a day, and 3+ times a day (F = 10.5, p < 0.001) and the Experience of Side Effect subscale distinguished between severity of somatization (F = 66.7, p < 0.001). The Weight Management subscale differentiated between weight loss groups (F = 9.8, p < 0.001). The Daily Life domain was not able to discriminate between having children or working status. This may be due to other factors, which overshadow the impact of children or work on daily life, such as stress. In a post-hoc analysis, the Daily Life domain was able to significantly differentiate based on degree of stress, which may be a more appropriate known group (F = 6.26, p < 0.01).

##### Interpretability

The total score and all domains met the MID threshold of 1/2 SD criteria as follows: Total (Δ = 8.5, 1/2 SD = 7.2); Weight Management (Δ = 11.6, 1/2 SD = 7.0); Treatment Burden (Δ = 13.1, 1/2 SD = 7.2); Experience of Side Effects (Δ = 14.6, 1/2 SD = 8.4); Psychological Health (Δ = 10.3, 1/2 SD = 10.4); and Daily Life (Δ = 16.1, 1/2 SD = 7.6) as shown in Table 3.

**Table 3:** Minimal Important Difference of the TRIM-Weight

	Mean	N	Mean	N	Difference	1/2 SD
**TRIM-Weight Domain**	**A little**		**Somewhat**			

Overall, how much does your weight loss medication impact your daily life?						
Daily Life	79.2 (15.2)	62	63.1 (21.9)	45	16.1	7.6

Overall, how well does your weight loss medication work? (reverse)						
	**A little**		**Somewhat**			
Weight Management	35.4 (15.5)	20	47.0 (13.9)	64	11.6	7.0

Overall, how convenient is your weight loss medication? (reverse)						
	**A little**		**Somewhat**			
Treatment Burden	41.7 (15.4)	15	54.8 (14.4)	38	13.1	7.2

Overall, how much do side effects from your weight loss medication negatively impact you?						
	**A little**		**Somewhat**			
Experience of Side Effects	66.3 (16.7)	59	51.7 (18.3)	45	14.6	8.4

Overall, how much does your weight loss medication negatively impact your psychological health?						
	**A little**		**Somewhat**			
Psychological Health	60.3 (20.9)	45	50.0 (20.4)	36	10.3	10.5

Overall, how well does your weight loss medication work?						
	**No impact at all**		**Slightly positive impact**			
TRIM-Weight Total	63.9 (21.1)	24	72.4 (14.4)	63	8.5	7.2

Finally, exploratory regression analyses were performed independently for each of the following variables on the TRIM-Weight Total Score: BMI category, gender, age and educational level. No significant relationships were found. When all variables were examined together in a final regression, gender was found to be significant (p < .000) with the impact of OAM being greater for women.

#### Final Measure

**Figure 2:**
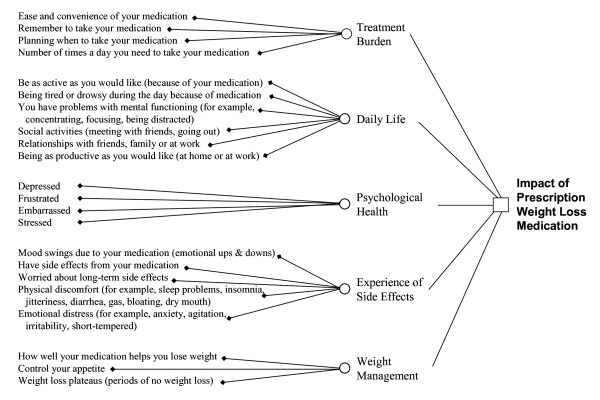
**Conceptual Framework**.

The validation process resulted in a 22-item TRIM-Weight. The conceptual framework identifying the relationship between items, domains, and the overall concept of the impact of prescription anti-obesity medications is shown in Figure 2.

Response burden was imputed from the respondent recorded time to complete the 43-item version TRIM-Weight of 6.60 (SD = 4.86) minutes. Total time was divided by 43 for a "per item time" and then the "per item time" was multiplied by 22. Thus, the time for completion of the 22-item TRIM-Weight is estimated at 3.38 (SD 2.49) minutes.

## Discussion

Patient reported outcomes can be understood either according to the broad stroke umbrella concept or as the individual domain components of that concept. Both are valid dimensions of a PRO measure and the appropriateness of the total versus the domain score is dependent upon the purpose for which the measure is being used. Therefore, the SAP for the TRIM-Weight was specifically written to validate the psychometric properties of both the total as well as domain subscale scores and the data from the validation study supports the claims for reliability and validity for both. As a result, each domain subscale can be used independently if assessment of that specific concept alone is required.

The conceptual model and the 5 domains impacted by AOM supported the TRIM-Weight item generation were developed based on direct patient input collected from focus groups and individual interviews. Each of these domains labelled Daily Life, Psychological Health, Weight Management, Treatment Burden and Experience of Side Effects are critical components of how patients experience AOM and are supported by previous research which has identified ways in which being overweight or obese adversely affects daily life and psychological health, including work productivity, attendance, social integration, overall psychological well being, stigmatization, self-esteem, joint pains, and depression [1,35]. In contrast, weight loss has led to increased participation in physical and social activities; greater energy and vitality; improvements in mood, self-confidence, self-concept, satisfaction with self-appearance and body image; decreased mirror avoidance; and improvements in emotional reaction, psychological stress, anxiety and depression [36-39].

The validation study was conducted via the web, which raises some potential bias in the sample selection for the study. However, we believe the bias introduced by a web-based study to be minimal, given the prevalence of computer access now available in the U.S., U.K., Canada and Australia. Also potentially biasing was the self-reported eligibility requirement of BMI and current AOM use. Given the minimal nature of the incentive to participate in the study, the fact that Survey Response subjects were pre-screened for eligibility before knowing the exact nature of the study and that Craig's List subjects were screened by telephone, we believe this bias was also not significant. The online format of the TRIM-Weight was exactly the same as a paper and pencil version, thus also suggesting that the two versions would be equivalent in psychometric properties [40-42]. Validation is an iterative process and future work should include the examination of psychometric properties in a placebo double blind trial design. Additionally, examining responsiveness using change in clinical parameters over time would be prudent.

As there were no longitudinal data available to fully examine the MID based on change over time, self-report items, one per domain of the TRIM-Weight, were used as anchors to approximate the MID. This analysis was considered exploratory and meant to provide preliminary estimates of differences established using an anchor-based approach. Since longitudinal data are not being used, one must be cautious in the interpretation of the results in relation to minimally important differences. As these findings should be considered preliminary, they should not be used as an estimation of the MID. However, they do indicate that an MID of 1/2 SD should be achievable for the TRIM-Weight.

The development of a PRO is an iterative process and a single PRO may truly never be validated for all possible uses. The goal of this first validation study was to determine the initial measurement model and fundamental reliability and validity of the TRIM-Weight. The cross sectional and web based nature of the study imposed certain limitations on the analyses which could be conducted. Future research examining criterion validity of the TRIM-Weight using clinical parameters, longitudinal data examining sensitivity to change and interpretability as well as scaling properties, and a confirmatory factor analysis derived from clinical trial data will be important next steps in the validation process.

Based on the clear negative impacts of AOM reported by the patients, it is evident that newer treatments that can reduce either the frequency or length of weight loss plateaus, continue to work over extended periods of time and allow for more consistent and long term weight loss without debilitating side effects, are needed. Improved understanding and assessment of the full range of these impacts on multiple dimensions of functioning and well-being will allow clinicians to realistically prepare patients for weight loss treatments, monitor impacts over time and adjust medications as needed to improve compliance.

## Conclusion

The development and validation of the Treatment Related Impact Measure-Weight (TRIM-Weight) has been conducted according to well-defined scientific principles for the creation of a PRO measure. Based on the evidence to date, it is suggested that the TRIM-Weight Total score, as well as each domain subscale, can be considered a brief, conceptually sound, rigorously developed PRO measure with strong evidence supporting the psychometric properties.

## Abbreviations

(AOM): anti-obesity medication; (TRIM-Weight): Treatment Related Impact Measure of Weight; (PRO): patient reported outcomes; (HRQoL): health related quality of life; (BMI): body mass index; (MID): minimally importance difference.

## Declaration of Competing interests

This study was funded by Novo Nordisk. Dr. Brod, Ms. Lessard and Mr. Bushnell are advisors/paid consultants to Novo Nordisk. Ms. Hammer and Ms. Kragh are employees of Novo Nordisk.

## Authors' contributions

MB was the lead contributor to the study design, instrument development and manuscript preparation and contributed to the data analysis and interpretation. MH contributed to the study design and manuscript preparation. NK contributed to the study design, instrument development, and manuscript preparation. SL contributed to the instrument development, data analysis and interpretation and manuscript preparation. DMB was the main contributor to the data analysis and interpretation and contributed to the manuscript preparation. All authors read and approved the final manuscript.
